# Real-world treatment durations, subsequent treatments, and switching of CDK4/6 inhibitors among patients with HR+/HER2− metastatic breast cancer

**DOI:** 10.1093/oncolo/oyag182

**Published:** 2026-05-07

**Authors:** Adam Brufsky, Rachel M Layman, Xianchen Liu, Benjamin Li, Lynn McRoy, Aaron Cohen, Melissa Estevez, Paul Cottu, Marc Thill, Giuseppe Curigliano, Hope S Rugo

**Affiliations:** Division of Hematology/Oncology, Department of Medicine, UPMC Hillman Cancer Center, University of Pittsburgh Medical Center, Pittsburgh, PA 15213, United States; Department of Breast Medical Oncology, The University of Texas MD Anderson Cancer Center, Houston, TX 77030, United States; US Oncology Medical Affairs, Pfizer Inc., New York, NY 10001, United States; Global Biometrics & Data Management, Pfizer Inc., New York, NY 10001, United States; US Oncology Medical Affairs, Pfizer Inc., New York, NY 10001, United States; Research Oncology, Flatiron Health Inc., New York, NY 10013, United States; Research Sciences, Flatiron Health Inc., New York, NY 10013, United States; Department of Medical Oncology & Institute of Women Cancers, Institut Curie, Université Paris Cité, Paris 75005, France; Department of Gynecology and Gynecological Oncology, Agaplesion Markus Krankenhaus, Klinik für Gynäkologie und Gynäkologische Onkologie, Brustzentrum, Frankfurt am Main 60431, Germany; Department of Oncology and Hemato-Oncology, University of Milano, Milano 20122, Italy; European Institute of Oncology, IRCCS, Milano 20141, Italy; Department of Medical Oncology, Division of Breast Medical Oncology, City of Hope Comprehensive Cancer Center, Duarte, CA 91010, United States

**Keywords:** abemaciclib, CDK4/6 inhibitor, metastatic breast cancer, palbociclib, ribociclib

## Abstract

**Background:**

Cyclin-dependent kinase 4/6 inhibitors (CDK4/6is), palbociclib, ribociclib, and abemaciclib, are approved for HR+/HER2− metastatic breast cancer (mBC). This real-world study evaluated treatment durations and subsequent treatments of patients with HR+/HER2− mBC who received CDK4/6is plus aromatase inhibitor (AI) in the United States.

**Methods:**

Adult patients with HR+/HER2− mBC who initiated first-line (1L) CDK4/6is plus AI (February 2015-July 2024) were selected from the US-based Flatiron Health Research database. Stabilized inverse probability of treatment weighting (sIPTW) was conducted. Kaplan−Meier analyses estimated treatment durations.

**Results:**

Of 11 557 patients, 8109, 2006, and 1442 received 1L palbociclib, ribociclib, and abemaciclib, respectively. After sIPTW, treatment duration was longer for the palbociclib group (median: 20.7 months) than the ribociclib (18.3 months) and abemaciclib (17.1 months) groups (ribociclib vs palbociclib: hazard ratio [HR] = 1.12 [95% confidence interval (CI), 1.05-1.20], *P *= .0008; abemaciclib vs palbociclib: HR = 1.13 [95% CI, 1.05-1.22], *P *= .0012). Treatment durations were similar between the ribociclib and abemaciclib groups (HR = 1.01 [95% CI, 0.92-1.11], *P *= .8280). Twelve-month treatment discontinuation rates were higher among patients initiating ribociclib or abemaciclib, at 39.4% and 41.1%, respectively, than among patients initiating palbociclib (33.3%). In the analysis of patients who initiated palbociclib, ribociclib, or abemaciclib from 2017 onward, 49.9%, 37.3%, and 39.4%, respectively, received subsequent treatments; CDK4/6i-containing regimens accounted for 42.3%, 54.1%, and 55.7%, respectively; notably, more patients initially treated with ribociclib or abemaciclib transitioned to palbociclib than those who switched oppositely.

**Conclusions:**

Treatment durations, discontinuation rates, and subsequent treatments differ between CDK4/6is for HR+/HER2− mBC in US routine clinical practice.

**Clinical Trial Registration Number:**

NCT06495164

Implications for Practice In this real-world study of over 11 500 patients with HR+/HER2− metastatic breast cancer (mBC) initiating first-line (1L) treatment with a CDK4/6 inhibitor (CDK4/6i) plus an aromatase inhibitor, those receiving palbociclib had longer treatment duration with lower discontinuation rates than patients initiating ribociclib or abemaciclib. CDK4/6i-containing regimens were the most commonly used subsequent treatment, with patients initially treated with ribociclib or abemaciclib more frequently switching to an alternative CDK4/6i, most often palbociclib. These findings highlight the importance of considering patient characteristics, efficacy, safety profiles, expected treatment duration, and potential subsequent therapies when selecting a CDK4/6i for 1L treatment of HR+/HER2− mBC.

## Introduction

A cyclin-dependent kinase 4/6 inhibitor (CDK4/6i) in combination with endocrine therapy (ET) is the standard of care for first-line (1L) treatment of hormone receptor-positive/human epidermal growth factor receptor 2-negative (HR+/HER2−) metastatic breast cancer (mBC).^1^^,^^2^ Currently, there are three CDK4/6is approved by the United States (US) Food and Drug Administration (FDA), including palbociclib, approved in 2015, and ribociclib and abemaciclib, both approved in 2017.^3–5^ In phase 3 randomized controlled trials (RCTs) of patients with HR+/HER2− mBC, 1L treatment with palbociclib (PALOMA-2), ribociclib (MONALEESA-2), or abemaciclib (MONARCH-3) in combination with an aromatase inhibitor (AI) significantly prolonged progression-free survival (PFS; primary endpoint) when compared with placebo plus AI, with similar reductions in the risk of disease progression seen across all three RCTs (range of hazard ratios [HR]: 0.54-0.57, all *P *< .0001).^6–8^ In these RCTs, median overall survival (OS; secondary endpoint) was numerically prolonged with palbociclib and abemaciclib versus control treatment, and ribociclib was shown to statistically significantly prolong OS.^9–11^ Several factors may contribute to the differences in efficacy in OS seen in the CDK4/6i versus ET RCTs, including study design, loss to follow-up and missing data, subsequent exposure to CDK4/6is, and patient characteristics (eg, poorer prognosis), in addition to true potential differences in efficacy in OS between the CDK4/6is.^12^^,^^13^

Presently, there are no published head-to-head RCTs that have assessed the comparative efficacy of CDK4/6is; however, several network meta-analyses and indirect treatment comparison studies that relied on data from the PALOMA, MONALEESA, and MONARCH RCTs and used different methodologies to account for the variability in the CDK4/6i RCTs have reported no significant differences in PFS and OS between the 3 CDK4/6is.^13–15^ Supporting this indirect clinical trial evidence, a growing body of real-world CDK4/6i comparative effectiveness studies have provided consistent findings, with no significant differences in real-world PFS (rwPFS) and/or OS observed across the CDK4/6is in combination with ET.^12^^,^^16–23^ For example, P-VERIFY (Palbociclib Verifying Evidence of Real-world Impact Study), a very large real-world study comparing the effectiveness of the 3 CDK4/6is in patients with HR+/HER2− mBC (*n* = 9146), provided evidence in US routine clinical practice that rwPFS and OS do not significantly differ across palbociclib, ribociclib, and abemaciclib when used in combination with an AI (rwPFS range of adjusted HRs [aHRs]: 0.96-0.98; OS range of aHRs: 0.95-0.98, all pairwise comparisons *P *> .05).^12^^,^^23^ However, PALMARES-2,^24^ a retrospective/prospective multicenter study conducted in Italy, and a retrospective nationwide database analysis conducted in Denmark^25^ have reported differences in rwPFS and/or OS among the three CDK4/6is. In PALMARES-2 (*n* = 1982), in the 1L setting, rwPFS was better among patients treated with ribociclib (aHR = 0.83 [95% CI, 0.73-0.95], *P *= .007) and abemaciclib (aHR = 0.76 [95% CI, 0.63-0.92], *P *= .004) versus palbociclib, but with no significant difference between abemaciclib and ribociclib (aHR = 0.91 [95% CI, 0.73-1.14], *P *= .425). Although immature in the PALMARES-2 study, the exploratory comparison of OS showed it was also prolonged with ribociclib (HR = 0.83 [95% CI, 0.81-0.84], *P *< .001) and abemaciclib (HR = 0.85 [95% CI, 0.74-0.97], *P *= .014) versus palbociclib.^24^ In the Danish study (1L *n* = 1554), rwPFS was also better in the 1L setting with ribociclib (aHR = 0.80 [95% CI, 0.68-0.96], *P *= .01) and abemaciclib (aHR = 0.74 [95% CI, 0.60-0.90], *P *= .005) versus palbociclib; however, OS in the 1L and rwPFS in the 2L were not significantly different across the three CDK4/6is.^25^ Several factors, including differences in sample sizes, patient characteristics, ET partners, follow-up times, assessments and definitions of outcomes, statistical approaches, CDK4/6i treatment duration, and subsequent treatments may have led to the inconsistent findings of these studies.^24^^,^^25^

While real-world comparative effectiveness studies of CDK4/6is in patients with HR+/HER2− mBC are increasing in number, there is relatively limited information on treatment durations and subsequent treatments of patients who receive 1L CDK4/6is. Therefore, in this large real-world study, we aimed to gain a better understanding of the differences in patient demographic and clinical characteristics, treatment durations, discontinuation rates, subsequent treatments, and switching between the three CDK4/6is in patients with HR+/HER2− mBC treated with a 1L CDK4/6i in combination with an AI in US routine clinical practice.

## Methods

### Study design

The current, large, real-world data analysis (NCT06495164) used the latest available Flatiron Health Research Database (data cutoff date: January 31, 2025) with more patients being included and having a longer follow-up than the prior OS and rwPFS analyses.^12^^,^^23^ The Flatiron Health Research Database is a deidentified electronic health record (EHR) database with over 750 000 patients with breast cancer represented in the EHR data at the time of this study. Structured and unstructured patient-level data are curated in this database using machine learning–enabled natural language processing and technology-enabled abstraction,^26–28^ with Flatiron Health’s quality and performance assessment frameworks enabling validation of study variables.^29–31^

Patients at least 18 years of age diagnosed with HR+/HER2− mBC who initiated palbociclib, ribociclib, or abemaciclib in combination with an AI between February 3, 2015 and July 31, 2024 (index period) as 1L treatment were included in the study population (Figure S1). The 1L treatment must have been initiated within 14 days before a patient’s mBC diagnosis date and up to 90 days after. Patients were excluded if they participated in a clinical trial during the study period. Patients were followed from the start of index treatment until the data cutoff date, death, or last medical activity, whichever occurred first.

This study was conducted in accordance with the Guidelines for Good Pharmacoepidemiology Practices issued by the International Society for Pharmacoepidemiology (ISPE), Good Practices for Outcomes Research issued by the International Society for Pharmacoeconomics and Outcomes Research (ISPOR), and Good Practices for Real-World Data Studies of Treatment and/or Comparative Effectiveness issued jointly by ISPOR and ISPE. As this study was a retrospective analysis of a deidentified database, it was exempt from institutional review board approval and did not require informed patient consent.

### CDK4/6i treatment durations and subsequent treatments

CDK4/6i treatment duration and discontinuation were assessed in all patients; subsequent treatments for mBC were examined in a separate analysis of patients who started 1L treatment from 2017 onward the 3 CDK4/6is were all approved in the US. A subsequent treatment was defined as a change in systemic therapy triggering a line advancement with the exception of changes in AI (ie, a change from one AI to another did not trigger a line advancement). However, a change to fulvestrant as the ET partner was considered as a line advancement regardless of whether the CDK4/6i remained the same or not. CDK4/6i switching was defined as changing the CDK4/6i with or without changing the AI partner. CDK4/6i treatment duration, regardless of the change of line of therapy (LOT), was defined as the time from the start of the specific CDK4/6i to treatment discontinuation or death, whichever came first. First-line CDK4/6i treatment duration was defined as the time from the start of the specific CDK4/6i to treatment discontinuation or death during the 1LOT. CDK4/6i discontinuation was defined as no medical records of the specific CDK4/6i use for at least 60 days after its last use during follow-up. Patients were censored at the earlier of their last medical activity or data cutoff if they were alive and had not experienced treatment discontinuation.

### Statistical analyses

Baseline demographic and clinical characteristics were stratified by index CDK4/6i treatment and summarized with descriptive statistics. Treatment durations with 95% confidence intervals (CIs) were estimated using the Kaplan–Meier method with Cox proportional hazards models used to calculate HRs and corresponding 95% CIs. Treatment durations were compared in the unadjusted analyses and after stabilized inverse probability treatment weighting (sIPTW; primary analysis). The sIPTW method was implemented to balance baseline demographic and clinical characteristics between CDK4/6i treatment groups. It used propensity scores estimated with a multivariable multinomial logistic regression model that incorporated baseline patient characteristic variables, including age, sex, race, practice type, Eastern Cooperative Oncology Group performance status, stage of disease at initial diagnosis, visceral metastasis, bone-only metastasis, number of metastatic disease sites, and disease-free interval (the time from initial diagnosis of breast cancer to diagnosis of mBC). A sensitivity analysis of duration of treatment was conducted with a multivariable Cox proportional hazards model that used these same variables as covariates. A separate analysis was carried out in patients who started 1L treatment from 2017 onward. All statistical analyses were performed using SAS version 9.4.

## Results

### Patients

A total of 11 557 patients with HR+/HER2− mBC who initiated 1L treatment with palbociclib (*n* = 8109), ribociclib (*n* = 2006), or abemaciclib (*n* = 1442) in combination with an AI between February 2015 and July 2024 were eligible for the study analyses (Figure S1). Demographic and clinical characteristics of patients in the unadjusted analysis and after sIPTW are shown in Table 1. In the unadjusted analysis, patients were older in the palbociclib group (median age: 67.0 years) than in the ribociclib (64.0 years) and abemaciclib (65.0 years) groups. Most patients across all study groups were female (∼99%). More patients in the ribociclib group (26.0%) were premenopausal than in the palbociclib (16.8%) and abemaciclib (22.4%) groups. Approximately one-third (33.7%-35.4%) of patients had visceral metastasis; slightly fewer patients in the abemaciclib group (42.5%) had bone-only metastasis than in the palbociclib (47.1%) and ribociclib (46.8%) groups. After sIPTW, baseline demographics and clinical characteristics were generally balanced between the three CDK4/6i groups. Arithmetic median durations of follow-up remained consistent before and after sIPTW, at 32 months for the palbociclib group, ∼16 months for the ribociclib group, and 20 months for the abemaciclib group.

**Table 1.** Demographic and clinical characteristics of patients in the unadjusted and sIPTW analyses.

Characteristic	Unadjusted analysis						After sIPTW					
	Cohort (*N* = 11 557)			Standardized difference^e^			Cohort (*N* = 11 555)			Standardized difference^e^		
	PAL + AI (*n *= 8109)	RIB + AI (*n *= 2006)	ABE + AI (*n *= 1442)	RIB + AI vs PAL + AI	ABE + AI vs PAL + AI	ABE + AI vs RIB + AI	PAL + AI (*n *= 8110)	RIB + AI (*n *= 2004)	ABE + AI (*n *= 1441)	RIB + AI vs PAL + AI	ABE + AI vs PAL + AI	ABE + AI vs RIB + AI
**Age at mBC diagnosis, years**												
Mean (SD)	65.6 (11.6)	62.8 (12.8)	63.4 (12.4)	−0.2316	−0.1882	0.0452	64.9 (11.9)	64.7 (12.1)	64.6 (12.1)	−0.0181	−0.0310	−0.0128
Median (IQR)	67.0 (17.0)	64.0 (19.0)	65.0 (18.0)				66.0 (17.0)	66.0 (17.0)	66.0 (17.0)			
**Age category, years**												
18-49	788 (9.7)	348 (17.3)	202 (14.0)	0.2245	0.1330	−0.0919	940 (11.6)	233 (11.6)	166 (11.5)	0.0006	−0.0029	−0.0034
50-64	2700 (33.3)	685 (34.1)	518 (35.9)	0.0180	0.0552	0.0372	2739 (33.8)	678 (33.8)	492 (34.1)	0.0012	0.0080	0.0068
65-74	2590 (31.9)	567 (28.3)	422 (29.3)	−0.0802	−0.0581	0.0221	2510 (30.9)	617 (30.8)	444 (30.8)	−0.0030	−0.0026	0.0005
≥ 75	2031 (25.0)	406 (20.2)	300 (20.8)	−0.1150	−0.1010	0.0140	1921 (23.7)	476 (23.8)	339 (23.5)	0.0015	−0.0039	−0.0055
Female sex, *n* (%)	8016 (98.9)	1985 (99.0)	1428 (99.0)	0.0096	0.0172	0.0076	8021 (98.9)	1982 (98.9)	1425 (98.9)	0.0028	−0.0029	−0.0057
**Menopausal status at initial diagnosis, *n* (%)**												
Postmenopausal	6226 (76.8)	1356 (67.6)	1026 (71.2)	−0.2060	−0.1285	0.0772	6090 (75.1)	1465 (73.1)	1063 (73.7)	−0.0458	−0.0314	0.0144
Premenopausal	1364 (16.8)	521 (26.0)	323 (22.4)	0.2245	0.1409	−0.0835	1493 (18.4)	418 (20.9)	287 (19.9)	0.0618	0.0380	−0.0238
Not documented	426 (5.3)	108 (5.4)	79 (5.5)	0.0058	0.0100	0.0042	437 (5.4)	100 (5.0)	76 (5.2)	−0.0189	−0.0069	0.0120
Male sex, *n* (%)	93 (1.1)	21 (1.0)	14 (1.0)	−0.0096	−0.0172	−0.0076	89 (1.1)	21 (1.1)	16 (1.1)	−0.0028	0.0029	0.0057
**Race, *n* (%)**												
White	5130 (63.3)	1206 (60.1)	818 (56.7)	−0.0647	−0.1337	−0.0689	5021 (61.9)	1240 (61.9)	892 (61.9)	−0.0003	0.0000	0.0003
Black	774 (9.5)	175 (8.7)	176 (12.2)	−0.0285	0.0855	0.1139	787 (9.7)	197 (9.8)	140 (9.7)	0.0034	−0.0007	−0.0041
Other	2205 (27.2)	625 (31.2)	448 (31.1)	0.0873	0.0854	−0.0019	2302 (28.4)	567 (28.3)	409 (28.4)	−0.0019	0.0005	0.0024
**Practice type, *n* (%)**												
Community	6726 (82.9)	1761 (87.8)	1207 (83.7)				6805 (83.9)	1688 (84.2)	1207 (83.7)			
Academic	1383 (17.1)	245 (12.2)	235 (16.3)	−0.1373	−0.0203	0.1170	1305 (16.1)	316 (15.8)	234 (16.3)	−0.0089	0.0046	0.0135
**Insurance type, *n* (%)**												
Commercial health plan plus any other	2621 (32.3)	574 (28.6)	435 (30.2)	−0.0806	−0.0465	0.0341	2569 (31.7)	607 (30.3)	445 (30.9)	−0.0297	−0.0168	0.0129
Commercial health plan	2562 (31.6)	778 (38.8)	580 (40.2)	0.1510	0.1806	0.0294	2593 (32.0)	754 (37.6)	572 (39.7)	0.1184	0.1616	0.0430
Medicare	482 (5.9)	77 (3.8)	42 (2.9)	−0.0977	−0.1478	−0.0513	464 (5.7)	87 (4.4)	45 (3.1)	−0.0624	−0.1257	−0.0640
Medicaid	124 (1.5)	29 (1.4)_	27 (1.9)	−0.0069	0.0265	0.0034	130 (1.6)	27 (1.3)	24 (1.6)	−0.0227	0.0028	0.0255
Other payer type	2320 (28.6)	548 (27.3)	358 (24.8)	−0.0288	−0.0856	−0.0568	2354 (29.0)	529 (26.4)	355 (24.6)	−0.0585	−0.0994	−0.0408
**Disease stage at initial diagnosis, *n* (%)**												
I	854 (10.5)	229 (11.4)	167 (11.6)	0.0283	0.0335	0.0052	879 (10.8)	218 (10.9)	158 (11.0)	0.0021	0.0038	0.0017
II	1706 (21.0)	425 (21.2)	270 (18.7)	0.0036	−0.0580	−0.0616	1687 (20.8)	422 (21.1)	301 (20.9)	0.0068	0.0021	−0.0047
III	840 (10.4)	186 (9.3)	155 (10.7)	−0.0365	0.0127	0.0492	830 (10.2)	205 (10.2)	145 (10.1)	−0.0007	−0.0052	−0.0045
IV	4282 (52.8)	1073 (53.5)	775 (53.7)	0.0137	0.0188	0.0051	4299 (53.0)	1055 (52.7)	763 (52.9)	−0.0070	−0.0011	0.0059
Not documented	427 (5.3)	93 (4.6)	75 (5.2)	−0.0290	−0.0029	0.0261	416 (5.1)	103 (5.2)	74 (5.1)	0.0015	0.0005	−0.0010
**ECOG PS, *n* (%)**												
0	2525 (31.1)	722 (36.0)	504 (35.0)	0.1029	0.0811	−0.0218	2631 (32.4)	651 (32.5)	464 (32.2)	0.0006	−0.0048	−0.0054
1	2191 (27.0)	558 (27.8)	379 (26.3)	0.0179	−0.0167	−0.0345	2196 (27.1)	539 (26.9)	390 (27.1)	−0.0036	−0.0003	0.0033
2, 3, or 4	1032 (12.7)	205 (10.2)	169 (11.7)	−0.0787	−0.0307	0.0480	986 (12.2)	242 (12.1)	176 (12.2)	−0.0030	0.0011	0.0041
Not documented	2361 (29.1)	521 (26.0)	390 (27.0)	−0.0704	−0.0461	0.0243	2297 (28.3)	572 (28.6)	411 (28.5)	0.0051	0.0045	−0.0006
**Disease-free interval, *n* (%)**												
De novo mBC	4282 (52.8)	1073 (53.5)	775 (53.7)	0.0137	0.0188	0.0051	4299 (53.0)	1055 (52.7)	763 (52.9)	−0.0070	−0.0011	0.0059
≤ 1 year	319 (3.9)	92 (4.6)	84 (5.8)	0.0323	0.0879	0.0558	346 (4.3)	86 (4.3)	61 (4.3)	0.0021	−0.0004	−0.0025
1-5 years	1236 (15.2)	276 (13.8)	219 (15.2)	−0.0421	−0.0015	0.0406	1216 (15.0)	302 (15.1)	215 (14.9)	0.0021	−0.0023	−0.0043
> 5 years	2272 (28.0)	565 (28.2)	364 (25.2)	0.0033	−0.0628	−0.0661	2250 (27.7)	561 (28.0)	402 (27.9)	0.0052	0.0033	−0.0020
Visceral metastasis^a^, *n* (%)	2730 (33.7)	684 (34.1)	511 (35.4)	0.0091	0.0372	0.0281	2752 (33.9)	676 (33.7)	488 (33.8)	−0.0039	−0.0019	0.0020
Bone-only metastasis^b^, *n* (%)	3819 (47.1)	938 (46.8)	613 (42.5)	−0.0067	−0.0923	−0.0856	3767 (46.5)	932 (46.5)	670 (46.5)	0.0012	0.0011	−0.0001
**Number of metastatic sites^c^, *n* (%)**												
1	4810 (59.3)	1159 (57.8)	811 (56.2)	−0.0313	−0.0623	−0.0310	4757 (58.6)	1174 (58.6)	845 (58.6)	−0.0013	−0.0011	0.0002
2	1769 (21.8)	483 (24.1)	323 (22.4)	0.0538	0.0141	−0.0397	1811 (22.3)	458 (22.8)	322 (22.4)	0.0123	0.0007	−0.0116
≥ 3	739 (9.1)	169 (8.4)	143 (9.9)	−0.0243	0.0274	0.0517	735 (9.1)	174 (8.7)	131 (9.1)	−0.0124	0.0000	0.0124
Not documented	791 (9.8)	195 (9.7)	165 (11.4)	−0.0011	0.0549	0.0560	808 (10.0)	198 (9.9)	144 (10.0)	−0.0034	0.0007	0.0042
**Number of metastatic sites among patients with ≥ 1 metastatic site^c^^, d^**												
Mean (SD)	1.5 (0.7)	1.5 (0.7)	1.5 (0.8)	0.0164	0.0552	0.0394	1.5 (0.7)	1.5 (0.7)	1.5 (0.7)	0.0015	0.0069	0.0054
Median (IQR)	1.0 (1.0)	1.0 (1.0)	1.0 (1.0)				1.0 (1.0)	1.0 (1.0)	1.0 (1.0)			
**Year of index date, *n* (%)**												
2015	519 (6.4)	0 (0)	0 (0)				515 (6.3)	0 (0)	0 (0)			
2016	792 (9.8)	0 (0)	0 (0)				794 (9.8)	0 (0)	0 (0)			
2017	845 (10.4)	71 (3.5)	0 (0)				845 (10.4)	74 (3.7)	0 (0)			
2018	915 (11.3)	148 (7.4)	69 (4.8)				917 (11.3)	148 (7.4)	69 (4.8)			
2019	1003 (12.4)	124 (6.2)	145 (10.1)				1003 (12.4)	121 (6.0)	146 (10.1)			
2020	1029 (12.7)	100 (5.0)	156 (10.8)				1037 (12.8)	95 (4.7)	155 (10.7)			
2021	1161 (14.3)	107 (5.3)	212 (14.7)				1160 (14.3)	100 (5.0)	209 (14.5)			
2022	976 (12.0)	255 (12.7)	283 (19.6)				977 (12.0)	251 (12.5)	288 (20.0)			
2023	605 (7.5)	702 (35.0)	363 (25.2)				600 (7.4)	718 (35.8)	359 (24.9)			
2024	264 (3.3)	499 (24.9)	214 (14.8)				262 (3.2)	498 (24.9)	216 (15.0)			
Median follow-up duration (IQR), months	32.1 (34.0)	16.7 (16.9)	20.1 (23.1)				32.1 (34.0)	16.4 (16.4)	20.1 (23.4)			

a Visceral disease is defined as metastatic disease in the lung and/or liver; patients could have had other sites of metastases.

b Bone-only disease is defined as metastatic disease in the bone only.

c Multiple metastases at the same site were counted as one site (eg, 3 bone metastases in the spine was considered only one site).

d Count of unique metastasic sites on or before index date regardless of the study period start date.

e The balance in these baseline characteristics was assessed using a standardized mean differences approach, with values ≥ 0.1 indicating a non-negligible imbalance. Abbreviations: ABE, abemaciclib; AI, aromatase inhibitor; ECOG PS, Eastern Cooperative Oncology Group performance status; IQR, interquartile range; mBC, metastatic breast cancer; PAL, palbociclib; RIB, ribociclib; SD, standard deviation; sIPTW, stabilized inverse probability of treatment weighting.

### Treatment durations and discontinuation rates

**Figure 1.**
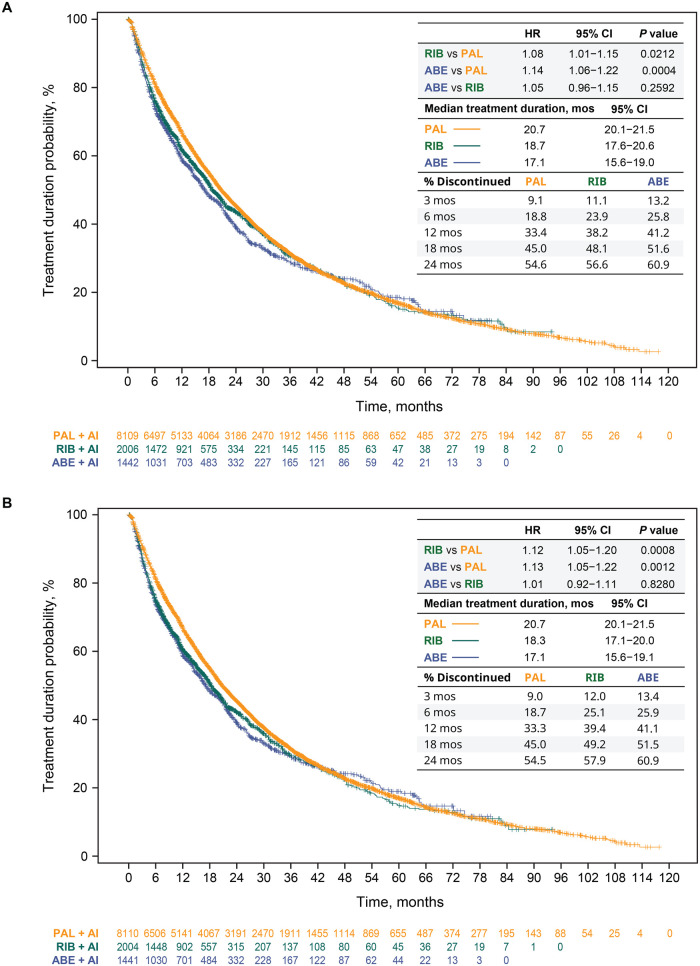
CDK4/6i treatment duration and discontinuation regardless of LOT change in the unadjusted analysis (A) and after sIPTW (B). Abbreviations: ABE, abemaciclib; AI, aromatase inhibitor; CDK4/6i, cyclin-dependent kinase 4/6 inhibitor; CI, confidence interval; HR, hazard ratio; LOT, line of therapy; mos, months; PAL, palbociclib; RIB, ribociclib; sIPTW, stabilized inverse probability of treatment weighting.

**Figure 2.**
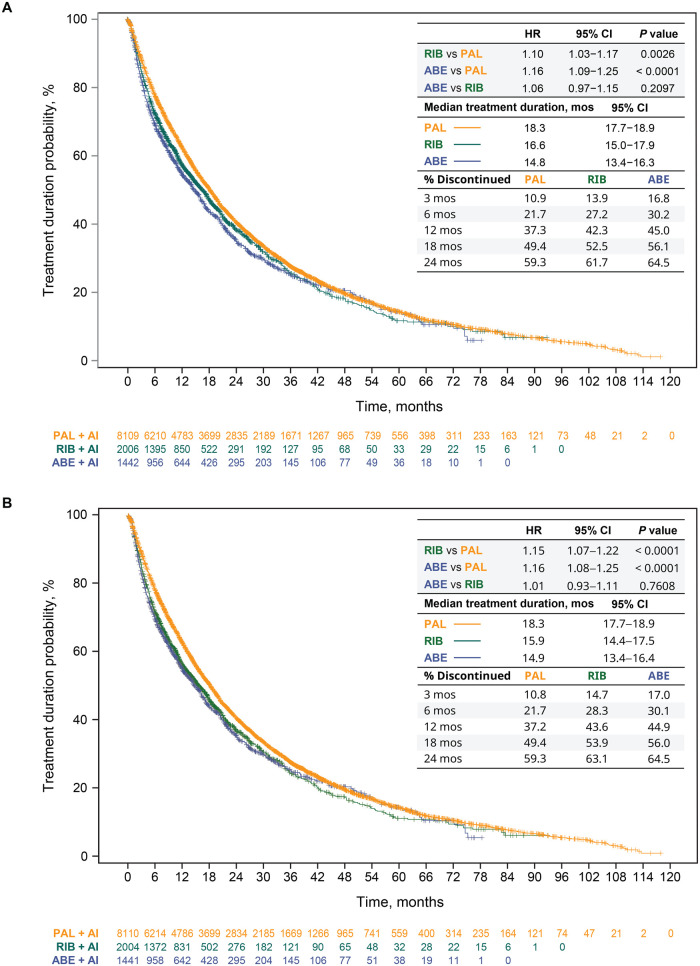
1L CDK4/6i treatment duration and discontinuation in the unadjusted analysis (A) and after sIPTW (B). Abbreviations: 1L, first-line; ABE, abemaciclib; AI, aromatase inhibitor; CDK4/6i, cyclin-dependent kinase 4/6 inhibitor; CI, confidence interval; HR, hazard ratio; mos, months; PAL, palbociclib; RIB, ribociclib; sIPTW, stabilized inverse probability of treatment weighting.

Median treatment durations are shown for the unadjusted analysis and after sIPTW in Figure 1A and 1B, respectively. In the primary analysis, after sIPTW, treatment duration, regardless of LOT change, was significantly longer for patients in the palbociclib group than in the ribociclib group (median = 20.7 [95% CI, 20.1-21.5] vs 18.3 [95% CI, 17.1-20.0] months; HR = 1.12 [95% CI, 1.05-1.20], *P *= .0008) and the abemaciclib group (median = 20.7 [95% CI, 20.1-21.5] vs 17.1 [95% CI, 15.6-19.1] months; HR = 1.13 [95% CI, 1.05-1.22], *P *= .0012) (Figure 1B). Median treatment durations were similar among the ribociclib and abemaciclib study groups (HR = 1.01 [95% CI, 0.92-1.11], *P *= .8280). These findings were consistent with those observed in the unadjusted (Figure 1A) and sensitivity analyses. In the sensitivity analysis, for example, the multivariable Cox proportional hazards model of treatment duration yielded an aHR of 1.10 (95% CI, 1.03-1.18, *P *= .0037) for ribociclib versus palbociclib; an aHR of 1.15 (95% CI, 1.07-1.23, *P *= .0002) for abemaciclib versus palbociclib; and an aHR of 1.04 (95% CI, 0.95-1.14, *P *= .3855) for abemaciclib versus ribociclib. Consistent results were also observed when duration of treatment was analyzed for CDK4/6i treatment in 1LOT only (Figure 2).

After sIPTW, compared with the palbociclib group, discontinuation rates were higher early on (3 months) among those who initiated ribociclib or abemaciclib (9.0% vs 12.0% or 13.4%, respectively), which remained consistent after 12 months (33.3% vs 39.4% or 41.1%, respectively) and 24 months (54.5% vs 57.9% or 60.9%, respectively) (Figure 1B). These findings were similar to those of the unadjusted analysis (Figure 1A).

### Analysis of patients who started index CDK4/6i plus AI treatment from 2017 onward

This analysis included all patients who initiated 1L CDK4/6i plus AI treatment from 2017 onward (total *N* = 10 246; 6798 with palbociclib, 2006 with ribociclib, 1442 with abemaciclib). Baseline demographics and clinical characteristics in the unadjusted analysis and after sIPTW for this patient population are shown in Table S1. After sIPTW, patient characteristics were generally balanced between the treatment groups.

#### Treatment durations and discontinuation rates

**Figure 3.**
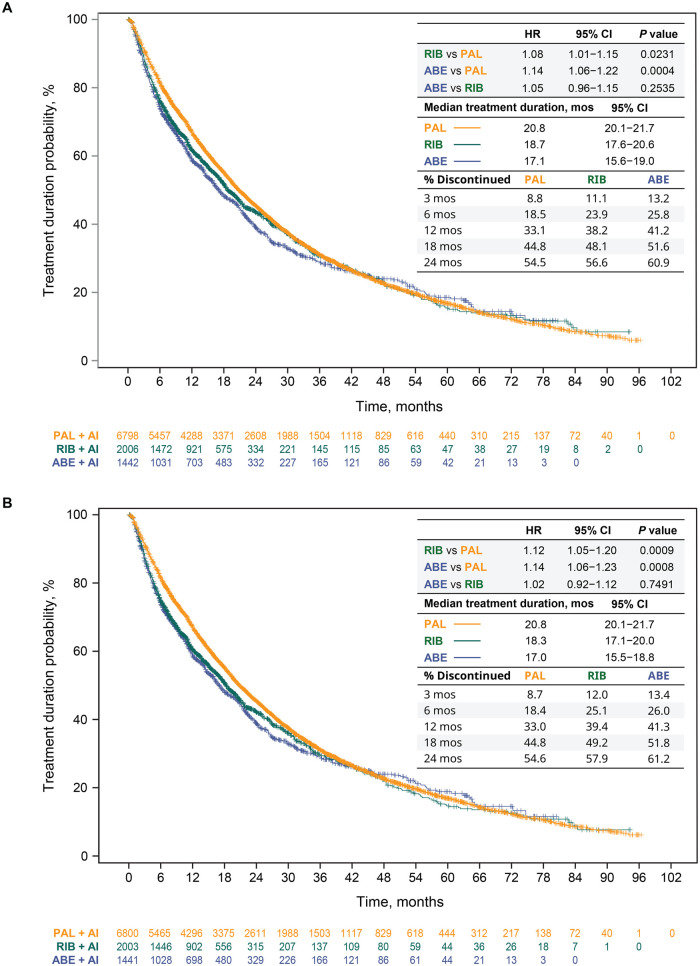
CDK4/6i treatment duration and discontinuation regardless of the LOT change in patients who started index treatment from 2017 onward in the unadjusted analysis (A) and after sIPTW (B). Abbreviations: ABE, abemaciclib; AI, aromatase inhibitor; CDK4/6i, cyclin-dependent kinase 4/6 inhibitor; CI, confidence interval; HR, hazard ratio; LOT, line of therapy; mos, months; PAL, palbociclib; RIB, ribociclib; sIPTW, stabilized inverse probability of treatment weighting.

**Figure 4.**
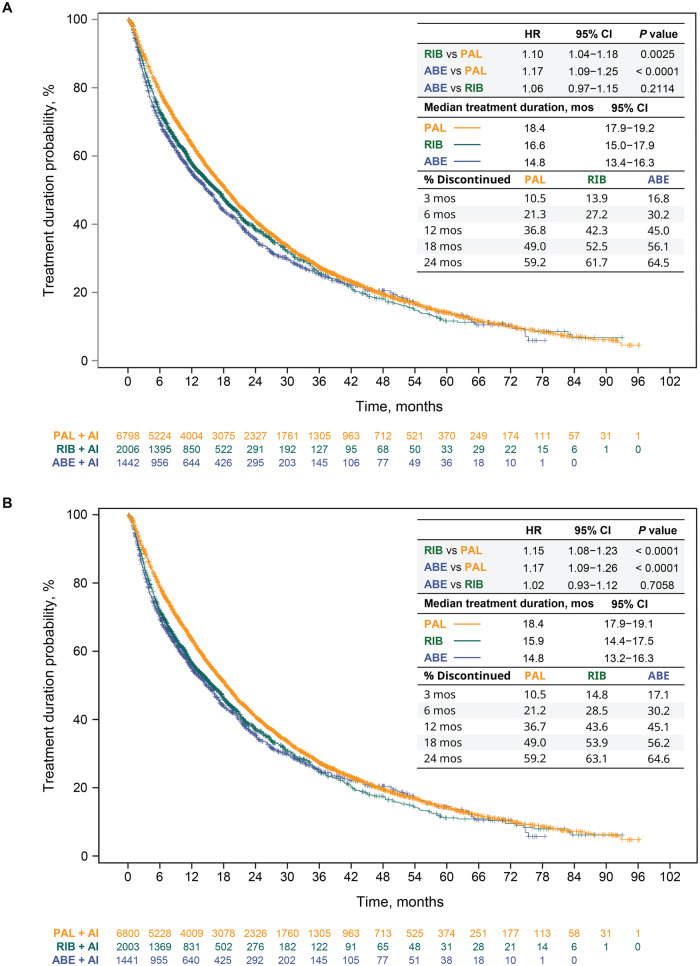
1L CDK4/6i treatment duration and discontinuation in patients who started index treatment from 2017 onward in the unadjusted analysis (A) and after sIPTW (B). Abbreviations: 1L, first-line; ABE, abemaciclib; AI, aromatase inhibitor; CDK4/6i, cyclin-dependent kinase 4/6 inhibitor; CI, confidence interval; HR, hazard ratio; mos, months; PAL, palbociclib; RIB, ribociclib; sIPTW, stabilized inverse probability of treatment weighting.

Treatment durations in this separate analysis for the unadjusted analysis (Figure 3A) and after sIPTW (Figure 3B) were consistent with those of the primary population. After sIPTW, treatment durations, regardless of LOT change, were significantly longer in the palbociclib (median = 20.8 months) than in the ribociclib (18.3 months) and abemaciclib (17.0 months) groups (ribociclib vs palbociclib: HR = 1.12 [95% CI, 1.05-1.20], *P *= .0009; abemaciclib vs palbociclib: HR = 1.14 [95% CI, 1.06-1.23], *P *= .0008). Median treatment durations were similar between the ribociclib and abemaciclib groups (HR = 1.02 [95% CI, 0.92-1.12], *P *= .7491). Similar findings were observed in the unadjusted analysis (Figure 3A) and sensitivity analysis. In the sensitivity analysis, for example, the multivariable Cox proportional hazards model of treatment duration yielded an aHR of 1.10 (95% CI, 1.03-1.18, *P *= .0037) for ribociclib versus palbociclib; an aHR of 1.15 (95% CI, 1.07-1.24, *P *= .0001) for abemaciclib versus palbociclib; and an aHR of 1.04 (95% CI, 0.95-1.14, *P *= .3692) for abemaciclib versus ribociclib. Consistent results were also observed when duration of treatment was analyzed for CDK4/6i treatment in the 1LOT only (Figure 4).

Discontinuation rates for patients who started 1L treatment from 2017 onward were also consistent with the findings of the primary population. After sIPTW (Figure 3B), compared with the palbociclib group, discontinuation rates were higher early on (3 months) among those who initiated ribociclib or abemaciclib (8.7% vs 12.0% or 13.4%, respectively), which remained consistent after 12 months (33.0% vs 39.4% or 41.3%, respectively) and 24 months (54.6% vs 57.9% or 61.2%, respectively).

#### Subsequent treatments and CDK4/6i switching

**Figure 5.**
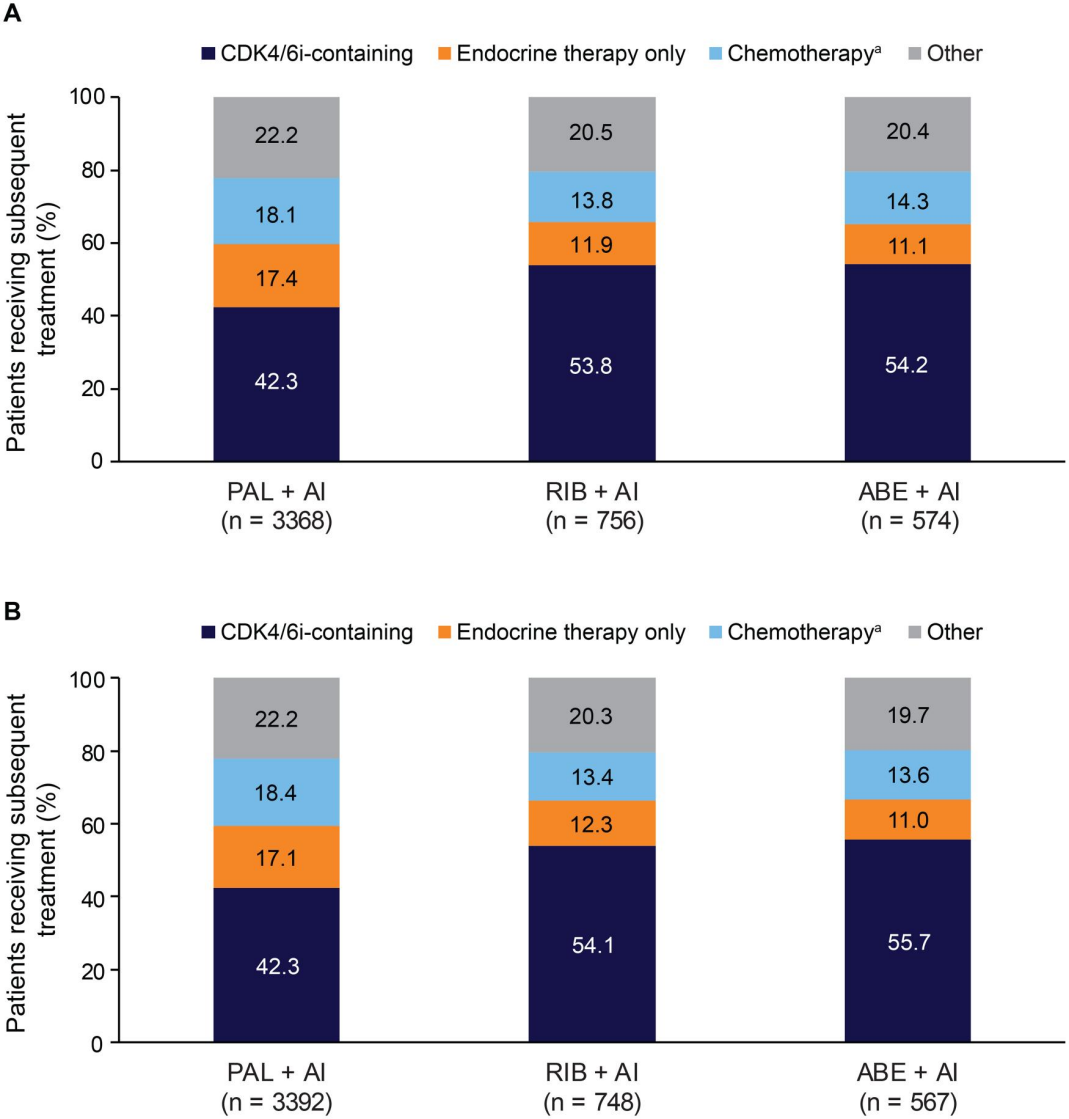
Subsequent treatments of patients who started index CDK4/6 plus AI treatment from 2017 onward in the unadjusted analysis (A) and after sIPTW (B). ^a^Chemotherapy with or without endocrine therapy. Percentage of patients who received subsequent treatment (defined as a change in systemic therapy triggering a line advancement with the exception of changes in AI). The percentage of patients who did not receive any subsequent treatment in the unadjusted analysis (A) were 50.5% in the PAL + AI group, 62.3% in the RIB + AI group, and 60.2% in the ABE + AI group with median durations of follow-up of 30.8 months, 16.7 months, and 20.1 months, respectively, and in the sIPTW analysis (B) were 50.1% in the PAL + AI group, 62.7% in the RIB + AI group, and 60.6% in the ABE + AI group with median durations of follow-up of 30.9 months, 16.4 months, and 20.1 months, respectively. Abbreviations: 1L, first-line; ABE, abemaciclib; AI, aromatase inhibitor; CDK4/6i, cyclin-dependent kinase 4/6 inhibitor; PAL, palbociclib; RIB, ribociclib; sIPTW, stabilized inverse probability of treatment weighting.

In the analysis of patients who started treatment from 2017 onward, after sIPTW, more patients treated with 1L palbociclib received subsequent treatment than those treated with ribociclib or abemaciclib (49.9% vs 37.3% or 39.4%, respectively; Table S2); however, follow-up time was longer for patients who received 1L palbociclib (median = 30.9 vs 16.4 vs 20.1 months, respectively; Table S1). Among the patients in the 1L palbociclib group who received subsequent treatment, 17.1% received ET alone, 18.4% received chemotherapy with or without ET, and 42.3% received a CDK4/6i-containing regimen; 22.2% received other therapies (Figure 5). Among the patients in the 1L ribociclib and abemaciclib groups who received subsequent treatments, similar proportions received ET alone (12.3% and 11.0%), chemotherapy with or without ET (13.4% and 13.6%), and CDK4/6i-containing treatment (54.1% and 55.7%), respectively.

**Figure 6.**
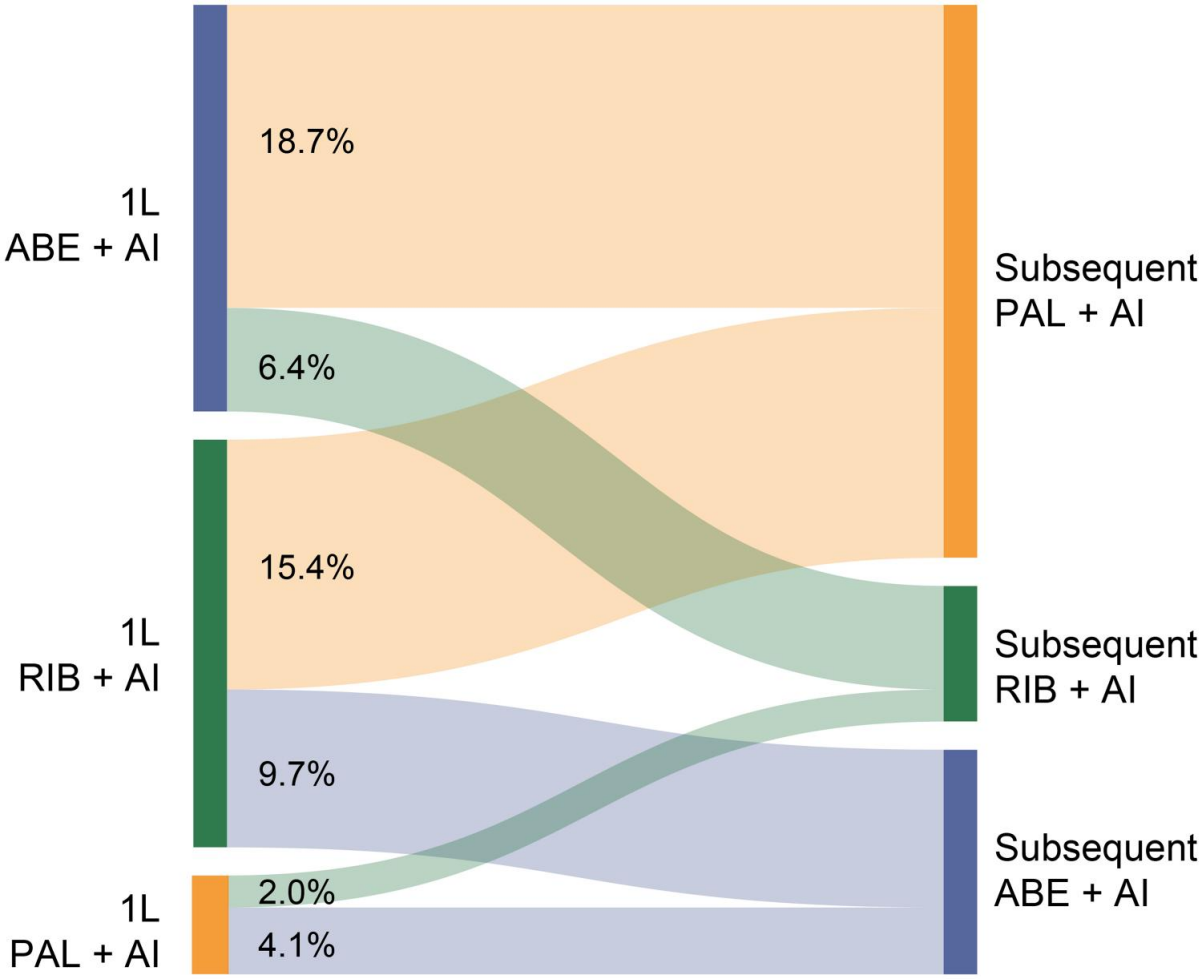
Sankey plot of CDK4/6i switching without changing of AI partner in patients who started index CDK4/6 plus AI treatment from 2017 onward after sIPTW. Percentages were calculated from patients with any subsequent treatment (index treatment: PAL + AI, *n* = 3392; RIB + AI, *n* = 748; ABE + AI, *n* = 567). Abbreviations: 1L, first-line; ABE, abemaciclib; AI, aromatase inhibitor; CDK4/6i, cyclin-dependent kinase 4/6 inhibitor; PAL, palbociclib; RIB, ribociclib; sIPTW, stabilized inverse probability of treatment weighting.

Among those who received subsequent treatment, the same CDK4/6i regardless of other anticancer treatment was continued for 25.8% of patients in the palbociclib group, 18.9% of patients in the ribociclib group, and 22.8% of patients in the abemaciclib group (Table S2). CDK4/6i switching rates of those who had any subsequent treatment, with or without changing their AI partner, were higher in the ribociclib group (20.3% switched to palbociclib; 14.1% switched to abemaciclib) and abemaciclib group (23.1% switched to palbociclib; 7.8% switched to ribociclib) than those in the palbociclib group (5.2% switched to ribociclib; 10.1% switched to abemaciclib). Of those who had any subsequent treatment, relatively few patients treated with 1L palbociclib switched to another CDK4/6i without changing the AI partner (6.1%), while approximately one-quarter of patients treated with either 1L ribociclib or abemaciclib switched to another CDK4/6i, most often to palbociclib (Figure 6). These findings were all generally consistent in the unadjusted analysis.

## Discussion

In this large real-world study, including 11 557 patients with HR+/HER2− mBC who received CDK4/6i plus AI treatment from February 2015 up until July 31, 2024, represented in the US-based EHR database, patients who initiated 1L palbociclib plus AI had significantly longer treatment duration than those who initiated 1L ribociclib or abemaciclib (sIPTW primary analysis: 20.7 vs 18.3 or 17.1 months, respectively). Additionally, in the 1L palbociclib group, rates of CDK4/6i discontinuation at 3, 6, 12, and 24 months were the lowest compared with the 1L ribociclib and abemaciclib groups. CDK4/6i-containing regimens were the most common subsequent treatments; patients treated with 1L ribociclib and abemaciclib more frequently switched to an alternative CDK4/6i, most often to palbociclib, than those treated with 1L palbociclib. These findings highlight the importance of considering patient characteristics, efficacy, safety profiles, expected treatment duration, and potential subsequent therapies when selecting a CDK4/6i for 1L treatment of HR+/HER2− mBC. Early detection of resistance and using biomarkers should be considered to guide and tailor subsequent treatment for patients with HR+/HER2− mBC.^32^^,^^33^

As a drug class, CDK4/6is have been most prominently associated with hematologic and gastrointestinal toxicity.^34^^,^^35^ However, while neutropenia, foremostly, but also anemia and thrombocytopenia are most frequently observed with palbociclib and, secondly, ribociclib, diarrhea, nausea, and/or vomiting more often occur in patients treated with abemaciclib and, secondly, ribociclib.^34^^,^^35^ Although less common, hepatic (eg, elevated liver enzymes), cardiac (eg, QT prolongation), and pulmonary (eg, interstitial lung disease) adverse events have also been found to occur at different rates with the three CDK4/6is.^34^^,^^35^ As the CDK4/6is differ in their toxicity profiles, ^34^^,^^35^ treatment durations and discontinuation rates could also have been differentially impacted by the management of the associated specific toxicities of each of the CDK4/6is, including any dose adjustments made in the real-world setting.

Real-world studies have reported widely varying rates, ranging from 33% to 81.5%, of dose reductions among patients with HR+/HER2− mBC treated with the CDK4/6is, palbociclib, ribociclib, and abemaciclib but have consistently seen that patients who are managed with dose reductions experience better survival outcomes.^36–39^ Three studies have also reported that treatment duration and/or time to next treatment are longer among those patients who have dose modifications compared with those who do not.^38–40^ Such findings suggest that dose modifications may be associated with increased tolerability, fewer adverse events and less discontinuation over the course of treatment.^38^^,^^39^ The longer treatment duration and less discontinuation seen with palbociclib in this study may be influenced by its longer time on the market and thus, greater provider experience with palbociclib treatment management. As information regarding dose adjustments were not available at the time of this study, it is important that future studies investigate how CDK4/6i dose modifications influence treatment duration. Dose optimization has become a highly recognized strategy for improving treatment outcomes, alongside personalizing patient care and emphasizing quality of life with both government (Project Optimus) and patient (Patient-Centered Dosing Initiative) -led initiatives.^41^ A patient advocate-led survey of 1221 patients with mBC has reported that among those who experienced at least one significant treatment-related side effect (86% of survey respondents), 46% had a dose reduction to manage it, and most (83%) reported “feeling better.”^42^ In this survey, 92% of patients affirmed they were willing to discuss flexible dosing options with their providers to maintain or improve their quality of life.^42^ These patient responses underscore that dose optimization is highly relevant considering patients with HR+/HER2− mBC may stay on a CDK4/6i for the rest of their life.

In this study, the most common subsequent treatments following 1L CDK4/6is were CDK4/6i-containing regimens (42.3%-55.7%); fewer patients received ET alone (11.0%-17.1%), chemotherapy with or without ET (13.4%-18.4%), or other therapies (19.7%-22.2%). Similar results were reported in another Flatiron study from Martin et al.,^43^ which specifically assessed systemic therapies following progression with a 1L CDK4/6i-containing treatment (*n* = 1210; 2015-2020; 88% of patients received 1L palbociclib). In the study, CDK4/6is were also the most common subsequent treatment, with 36% of patients continuing on a CDK4/6i, a proportion that increased over the study years; 29.7% received chemotherapy (proportion decreased over study years), 12.4% received ET alone, and 20.2% received other therapies.^43^ Furthermore, the study found that 74.4% of patients continued on the same CDK4/6i, with more patients treated with 1L ribociclib and abemaciclib having switched to different CDK4/6is than those treated with 1L palbociclib.^43^ This was also true in our study, with the majority of patients who received 1L palbociclib who went on to receive a subsequent treatment continuing with palbociclib; however, those who received 1L ribociclib and abemaciclib more frequently switched to another CDK4/6i, most often palbociclib. More frequent switching of CDK4/6is in patients treated with ribociclib and abemaciclib was also seen in the PALMARES-2 study; the primary reason for switching CDK4/6is was treatment-induced toxicity (86%).^24^ Finally, our results were also similar to a small study of patients with HR+/HER2− mBC treated with palbociclib plus an AI (*n* = 242) that used the US Syapse Learning Health Network longitudinal database (2015-2020), in which median 1L treatment duration was 23.9 months and CDK4/6is were the most common subsequent treatment (39.6%) with 73.8% receiving palbociclib, while 20.8% received chemotherapy.^44^ While studies conducted in the US show relatively similar treatment sequencing, those conducted in other countries indicate that continuing CDK4/6is is less common, which may be related to guidelines not recommending rechallenging patients with another CDK4/6i.^45^^,^^46^

In the phase 2 MAINTAIN clinical trial, improved PFS was seen among patients with HR+/HER2− mBC who had progressed on a CDK4/6i and then switched to ribociclib plus ET versus ET alone (*n* = 119; median PFS: 5.3 vs 2.8 months, HR = 0.57 [95% CI, 0.39-0.85], *P *= .006).^47^ The phase 3 post-MONARCH clinical trial also reported improved PFS among patients with HR+/HER2− mBC who had progressed on a CDK4/6i plus AI and then switched to abemaciclib plus fulvestrant versus placebo plus fulvestrant (*n* = 368; median PFS: 6.0 vs 5.3 months, HR = 0.73 [95% CI, 0.57-0.95], *P *= .017).^48^ However, no improvement in PFS compared with ET alone was found in the PACE and PALMIRA trials of patients whose disease had progressed on a CDK4/6i plus ET and then switched to or were rechallenged with palbociclib plus ET.^49^^,^^50^ Further research remains a critical need to understand the association of survival outcomes with subsequent treatments, patient characteristics (eg comorbidities), and gene mutations over the course of CDK4/6i treatment.

Key strengths of our study include that, to our knowledge, it is the largest study to date of 1L CDK4/6i treatment durations, subsequent treatments, and CDK4/6i switching among patients with HR+/HER2− mBC, the diversity of patients in demographic and clinical characteristics represented in the analyses, and the comprehensiveness of longitudinal data collected from US routine clinical practice and represented in the data source. Additionally, multiple baseline demographic and clinical characteristics were adjusted using sIPTW or multivariable regression analysis, with the EHR-derived data in the real-world database having been validated using quality and performance assessment frameworks, as described previously.^29–31^ Furthermore, the robustness of our findings was evidenced by their consistency across multiple methodologies, including after sIPTW (primary analysis), multivariable (sensitivity analysis) adjustment and in the analysis of patients treated from 2017 onward.

Limitations of this study include that it was a retrospective database analysis of EHRs, which may have inaccurate or incomplete data capture. Also, information about dose adjustments or reasons for discontinuation and subsequent treatments (eg, disease progression or toxicity) were not available in the data source at the time of data analysis, and further study is warranted. Although sIPTW and multivariable analyses were utilized to mitigate potential bias in this study, the effects of unmeasured covariates, such as socioeconomic factors and prior adjuvant therapies, could not be excluded. Compared with the palbociclib group, the ribociclib and abemaciclib groups had smaller sample sizes and shorter follow-up durations. Finally, our findings may not generalize to patient populations that are underrepresented in the data source, as well as to those in other countries.

## Conclusions

In this large real-world study (*n* = 11 557), patients with HR+/HER2− mBC who initiated 1L palbociclib in combination with an AI had longer treatment duration with lower discontinuation rates compared with those who initiated 1L ribociclib or abemaciclib. CDK4/6i-containing regimens were the most commonly used subsequent treatments. Further research is warranted to understand the clinical outcomes associated with early discontinuation and switches between CDK4/6is among patients with HR+/HER2− mBC.

## Supplementary Material

oyag182_Supplementary_Data

## Data Availability

The data that support the findings of this study were originated by and are property of Flatiron Health, Inc., which has restrictions prohibiting the authors from making the data set publicly available. Requests for data sharing by license or by permission for the specific purpose of replicating results in this manuscript can be submitted to PublicationsDataAccess@flatiron.com.
