# Supplementary material for: Real-world treatment durations, subsequent treatments, and switching of CDK4/6 inhibitors among patients with HR+/HER2− metastatic breast cancer
Source: Oncologist. 2026 May 7;31(6):oyag182. doi: 10.1093/oncolo/oyag182 (PMC13228134; doi:10.1093/oncolo/oyag182)
Supplement: oyag182_Supplementary_Data [file oyag182_Supplementary_Data.docx]

# Supplementary Material

**Supplementary Table S1. Demographics and clinical characteristics of patients who started index CDK4/6i plus AI treatment from 2017 and onward in the unadjusted and sIPTW analyses**

| **Characteristic** | **Unadjusted analysis** | | | | | | **After sIPTW** | | | | | |
| --- | --- | --- | --- | --- | --- | --- | --- | --- | --- | --- | --- | --- |
|  | **Cohort (N = 10,246)** | | | **Standardized difference^e^** | | | **Cohort (N = 10,244)** | | | **Standardized difference^e^** | | |
|  | **PAL + AI**  **(*n* = 6798)** | **RIB + AI**  **(*n* = 2006)** | **ABE + AI**  **(*n* = 1442)** | **RIB + AI vs**  **PAL + AI** | **ABE + AI vs**  **PAL + AI** | **ABE + AI vs**  **RIB + AI** | **PAL + AI**  **(*n* = 6800)** | **RIB + AI**  **(*n* = 2003)** | **ABE + AI**  **(*n* = 1441)** | **RIB + AI vs**  **PAL + AI** | **ABE + AI vs**  **PAL + AI** | **ABE + AI vs**  **RIB + AI** |
| Age at mBC diagnosis, years | | | | | | |  |  |  |  |  |  |
| Mean (SD) | 66.0 (11.6) | 62.8 (12.8) | 63.4 (12.4) | -0.2589 | -0.2160 | 0.0452 | 65.1 (12.0) | 64.8 (12.1) | 64.7 (12.1) | -0.0209 | -0.0333 | -0.0124 |
| Median (IQR) | 67.0 (16.0) | 64.0 (19.0) | 65.0 (18.0) |  |  |  | 66.0 (16.0) | 66.0 (17.0) | 66.0 (17.0) |  |  |  |
| Age category, years | | | | | | |  |  |  |  |  |  |
| 18−49 | 634 (9.3) | 348 (17.3) | 202 (14.0) | 0.2376 | 0.1462 | -0.0919 | 788 (11.6) | 232 (11.6) | 166 (11.5) | 0.0004 | -0.0030 | -0.0034 |
| 50−64 | 2220 (32.7) | 685 (34.1) | 518 (35.9) | 0.0316 | 0.0688 | 0.0372 | 2273 (33.4) | 672 (33.6) | 487 (33.8) | 0.0031 | 0.0088 | 0.0057 |
| 65−74 | 2154 (31.7) | 567 (28.3) | 422 (29.3) | -0.0747 | -0.0526 | 0.0221 | 2083 (30.6) | 609 (30.4) | 439 (30.5) | -0.0044 | -0.0030 | 0.0014 |
| ≥ 75 | 1790 (26.3) | 406 (20.2) | 300 (20.8) | -0.1445 | -0.1305 | 0.0140 | 1656 (24.4) | 489 (24.4) | 348 (24.2) | 0.0010 | -0.0043 | -0.0053 |
| Female sex, *n* (%) | 6712 (98.7) | 1985 (99.0) | 1428 (99.0) | 0.0204 | 0.0280 | 0.0076 | 6721 (98.8) | 1980 (98.8) | 1424 (98.8) | 0.0014 | -0.0005 | -0.0020 |
| Menopausal status at initial diagnosis, *n* (%) | | | | | | |  |  |  |  |  |  |
| Postmenopausal | 5239 (77.1) | 1356 (67.6) | 1026 (71.2) | -0.2129 | -0.1354 | 0.0772 | 5098 (75.0) | 1465 (73.1) | 1064 (73.9) | -0.0418 | -0.0253 | 0.0165 |
| Premenopausal | 1123 (16.5) | 521 (26.0) | 323 (22.4) | 0.2326 | 0.1489 | -0.0835 | 1260 (18.5) | 416 (20.8) | 285 (19.8) | 0.0564 | 0.0321 | -0.0243 |
| Not documented | 350 (5.1) | 108 (5.4) | 79 (5.5) | 0.0105 | 0.0147 | 0.0042 | 364 (5.3) | 99 (5.0) | 75 (5.2) | -0.0177 | -0.0073 | 0.0103 |
| Male sex, *n* (%) | 86 (1.3) | 21 (1.0) | 14 (1.0) | -0.0204 | -0.0280 | -0.0076 | 80 (1.2) | 23 (1.2) | 17 (1.2) | -0.0014 | 0.0005 | 0.0020 |
| Race, *n* (%) | | | | | | |  |  |  |  |  |  |
| White | 4231 (62.2) | 1206 (60.1) | 818 (56.7) | -0.0435 | -0.1125 | -0.0689 | 4152 (61.1) | 1222 (61.0) | 879 (61.0) | -0.0011 | -0.0002 | 0.0009 |
| Black | 665 (9.8) | 175 (8.7) | 176 (12.2) | -0.0365 | 0.0775 | 0.1139 | 672 (9.9) | 200 (10.0) | 143 (9.9) | 0.0030 | 0.0006 | -0.0025 |
| Other | 1902 (28.0) | 625 (31.2) | 448 (31.1) | 0.0697 | 0.0678 | -0.0019 | 1977 (29.1) | 581 (29.0) | 419 (29.1) | -0.0008 | -0.0001 | 0.0007 |
| Practice type, *n* (%) | | | | | | |  |  |  |  |  |  |
| Community | 5668 (83.4) | 1761 (87.8) | 1207 (83.7) |  |  |  | 5736 (84.3) | 1698 (84.8) | 1212 (84.2) |  |  |  |
| Academic | 1130 (16.6) | 245 (12.2) | 235 (16.3) | -0.1258 | -0.0088 | 0.1170 | 1065 (15.7) | 305 (15.2) | 228 (15.8) | -0.0125 | 0.0051 | 0.0175 |
| Insurance type, *n* (%) | | | | | | |  |  |  |  |  |  |
| Commercial health plan plus any other | 2216 (32.6) | 574 (28.6) | 435 (30.2) | -0.0865 | -0.0524 | 0.0341 | 2161 (31.8) | 605 (30.2) | 445 (30.9) | -0.0336 | -0.0194 | 0.0142 |
| Commercial health plan | 2213 (32.6) | 778 (38.8) | 580 (40.2) | 0.1303 | 0.1599 | 0.0294 | 2245 (33.0) | 753 (37.6) | 573 (39.8) | 0.0964 | 0.1412 | 0.0447 |
| Medicare | 397 (5.8) | 77 (3.8) | 42 (2.9) | -0.0934 | -0.1435 | -0.0513 | 378 (5.6) | 88 (4.4) | 46 (3.2) | -0.0548 | -0.1173 | -0.0632 |
| Medicaid | 109 (1.6) | 29 (1.4) | 27 (1.9) | -0.0129 | 0.0206 | 0.0334 | 116 (1.7) | 27 (1.3) | 24 (1.6) | -0.0308 | -0.0049 | 0.0260 |
| Other payer type | 1863 (27.4) | 548 (27.3) | 358 (24.8) | -0.0020 | -0.0587 | -0.0568 | 1901 (27.9) | 530 (26.5) | 353 (24.5) | -0.0333 | -0.0779 | -0.0446 |
| Disease stage at initial diagnosis, *n* (%) | | | | | | |  |  |  |  |  |  |
| I | 725 (10.7) | 229 (11.4) | 167 (11.6) | 0.0240 | 0.0291 | 0.0052 | 745 (11.0) | 219 (10.9) | 160 (11.1) | -0.0005 | 0.0041 | 0.0046 |
| II | 1427 (21.0) | 425 (21.2) | 270 (18.7) | 0.0048 | -0.0569 | -0.0616 | 1410 (20.7) | 420 (21.0) | 298 (20.7) | 0.0062 | -0.0006 | -0.0068 |
| III | 661 (9.7) | 186 (9.3) | 155 (10.7) | -0.0154 | 0.0338 | 0.0492 | 667 (9.8) | 197 (9.8) | 139 (9.6) | 0.0012 | -0.0060 | -0.0073 |
| IV | 3613 (53.1) | 1073 (53.5) | 775 (53.7) | 0.0068 | 0.0120 | 0.0051 | 3621 (53.2) | 1059 (52.9) | 767 (53.3) | -0.0071 | 0.0005 | 0.0076 |
| Not documented | 372 (5.5) | 93 (4.6) | 75 (5.2) | -0.0382 | -0.0121 | 0.0261 | 358 (5.3) | 107 (5.3) | 76 (5.3) | 0.0036 | 0.0021 | -0.0015 |
| ECOG PS, *n* (%) | | | | | | |  |  |  |  |  |  |
| 0 | 2207 (32.5) | 722 (36.0) | 504 (35.0) | 0.0744 | 0.0526 | -0.0218 | 2276 (33.5) | 672 (33.5) | 478 (33.2) | 0.0016 | -0.0058 | -0.0074 |
| 1 | 1876 (27.6) | 558 (27.8) | 379 (26.3) | 0.0049 | -0.0296 | -0.0345 | 1868 (27.5) | 547 (27.3) | 396 (27.5) | -0.0034 | 0.0006 | 0.0040 |
| 2, 3, or 4 | 924 (13.6) | 205 (10.2) | 169 (11.7) | -0.1043 | -0.0563 | 0.0480 | 861 (12.7) | 253 (12.6) | 183 (12.7) | -0.0016 | 0.0016 | 0.0033 |
| Not documented | 1791 (26.3) | 521 (26.0) | 390 (27.0) | -0.0085 | 0.0158 | 0.0243 | 1795 (26.4) | 531 (26.5) | 383 (26.6) | 0.0030 | 0.0043 | 0.0014 |
| Disease-free interval, *n* (%) | | | | | | |  |  |  |  |  |  |
| De novo mBC | 3613 (53.1) | 1073 (53.5) | 775 (53.7) | 0.0068 | 0.0120 | 0.0051 | 3621 (53.2) | 1059 (52.9) | 767 (53.3) | -0.0071 | 0.0005 | 0.0076 |
| ≤ 1 year | 279 (4.1) | 92 (4.6) | 84 (5.8) | 0.0236 | 0.0793 | 0.0558 | 301 (4.4) | 89 (4.5) | 64 (4.4) | 0.0016 | -0.0001 | -0.0018 |
| 1−5 years | 1003 (14.8) | 276 (13.8) | 219 (15.2) | -0.0285 | 0.0121 | 0.0406 | 995 (14.6) | 296 (14.8) | 210 (14.5) | 0.0045 | -0.0025 | -0.0071 |
| > 5 years | 1903 (28.0) | 565 (28.2) | 364 (25.2) | 0.0038 | -0.0623 | -0.0661 | 1884 (27.7) | 558 (27.9) | 400 (27.8) | 0.0036 | 0.0015 | -0.0021 |
| Visceral metastasis^a^, *n* (%) | 2288 (33.7) | 684 (34.1) | 511 (35.4) | 0.0093 | 0.0374 | 0.0281 | 2311 (34.0) | 678 (33.9) | 490 (34.0) | -0.0023 | 0.0005 | 0.0028 |
| Bone-only metastasis^b^, *n* (%) | 3190 (46.9) | 938 (46.8) | 613 (42.5) | -0.0033 | -0.0889 | -0.0856 | 3144 (46.2) | 927 (46.3) | 665 (46.2) | 0.0012 | -0.0013 | -0.0025 |
| Number of metastatic sites^c^, *n* (%) | | | | | | |  |  |  |  |  |  |
| 1 | 4036 (59.4) | 1159 (57.8) | 811 (56.2) | -0.0324 | -0.0634 | -0.0310 | 3983 (58.6) | 1172 (58.5) | 841 (58.4) | -0.0009 | -0.0037 | -0.0029 |
| 2 | 1490 (21.9) | 483 (24.1) | 323 (22.4) | 0.0513 | 0.0116 | -0.0397 | 1529 (22.5) | 459 (22.9) | 325 (22.6) | 0.0109 | 0.0023 | -0.0087 |
| ≥ 3 | 611 (9.0) | 169 (8.4) | 143 (9.9) | -0.0200 | 0.0318 | 0.0517 | 609 (9.0) | 174 (8.7) | 130 (9.0) | -0.0104 | 0.0014 | 0.0118 |
| Not documented | 661 (9.7) | 195 (9.7) | 165 (11.4) | -0.0001 | 0.0559 | 0.0560 | 679 (10.0) | 197 (9.9) | 144 (10.0) | -0.0041 | 0.0016 | 0.0057 |
| Number of metastatic sites among patients with ≥ 1 metastatic site^c,d^ | | | | | | |  |  |  |  |  |  |
| Mean (SD) | 1.5 (0.7) | 1.5 (0.7) | 1.5 (0.8) | 0.0182 | 0.0570 | 0.0394 | 1.5 (0.7) | 1.5 (0.7) | 1.5 (0.7) | 0.0016 | 0.0082 | 0.0066 |
| Median (IQR) | 1.0 (1.0) | 1.0 (1.0) | 1.0 (1.0) |  |  |  | 1.0 (1.0) | 1.0 (1.0) | 1.0 (1.0) |  |  |  |
| Year of index date, *n* (%) | | | | | | |  |  |  |  |  |  |
| 2017 | 845 (12.4) | 71 (3.5) | 0 (0) |  |  |  | 851 (12.5) | 72 (3.6) | 0 (0) |  |  |  |
| 2018 | 915 (13.5) | 148 (7.4) | 69 (4.8) |  |  |  | 920 (13.5) | 147 (7.3) | 68 (4.7) |  |  |  |
| 2019 | 1003 (14.8) | 124 (6.2) | 145 (10.1) |  |  |  | 1003 (14.8) | 121 (6.1) | 146 (10.1) |  |  |  |
| 2020 | 1029 (15.1) | 100 (5.0) | 156 (10.8) |  |  |  | 1038 (15.3) | 95 (4.7) | 155 (10.7) |  |  |  |
| 2021 | 1161 (17.1) | 107 (5.3) | 212 (14.7) |  |  |  | 1156 (17.0) | 101 (5.0) | 209 (14.5) |  |  |  |
| 2022 | 976 (14.4) | 255 (12.7) | 283 (19.6) |  |  |  | 975 (14.3) | 248 (12.4) | 286 (19.8) |  |  |  |
| 2023 | 605 (8.9) | 702 (35.0) | 363 (25.2) |  |  |  | 596 (8.8) | 717 (35.8) | 360 (25.0) |  |  |  |
| 2024 | 264 (3.9) | 499 (24.9) | 214 (14.8) |  |  |  | 261 (3.8) | 502 (25.1) | 217 (15.1) |  |  |  |
| Median follow-up duration (IQR), months | 30.8 (31.7) | 16.7 (16.9) | 20.1 (23.1) |  |  |  | 30.9 (31.7) | 16.4 (16.2) | 20.1 (23.3) |  |  |  |

^a^ Visceral disease is defined as metastatic disease in the lung and/or liver; patients could have had other sites of metastases.
^b^ Bone-only disease is defined as metastatic disease in the bone only.
^c^ Multiple metastases at the same site were counted as one site (eg, 3 bone metastases in the spine was considered only one site).

^d^ Count of unique metastasic sites on or before index date regardless of the study period start date.

^e^ The balance in these baseline characteristics was assessed using a standardized mean differences approach, with values ≥ 0.1 indicating a non-negligible imbalance. Abbreviations: ABE, abemaciclib; AI, aromatase inhibitor; CDK4/6i, cyclin-dependent kinase 4/6 inhibitor; ECOG PS, Eastern Cooperative Oncology Group performance status; IQR, interquartile range; mBC, metastatic breast cancer; PAL, palbociclib; RIB, ribociclib; SD, standard deviation; sIPTW, stabilized inverse probability of treatment weighting.

**Supplementary Table S2. Subsequent CDK4/6i treatments and switching in patients who started index treatment from 2017 and onward in the unadjusted analysis and after sIPTW**

| **Unadjusted analysis** | | **Subsequent CDK4/6i treatment and switching, n (%^a^)** | | | | | |
| --- | --- | --- | --- | --- | --- | --- | --- |
| **1L CDK4/6i + AI** | **Any subsequent treatment**  **n (%)** | PAL-containing | PAL + AI^b^ | RIB-containing | RIB + AI^b^ | ABE-containing | ABE + AI^b^ |
| **1L PAL + AI**  **(n = 6798)** | 3368 (49.5) | 872 (25.9) | NA | 172 (5.1) | 66 (2.0) | 338 (10.0) | 140 (4.2) |
| **1L RIB + AI**  **(n = 2006)** | 756 (37.7) | 151 (20.0) | 113 (14.9) | 147 (19.4) | NA | 104 (13.8) | 70 (9.3) |
| **1L ABE + AI**  **(n = 1442)** | 574 (39.8) | 125 (21.8) | 99 (17.2) | 43 (7.5) | 36 (6.3) | 132 (23.0) | NA |
| **After sIPTW** | | **Subsequent CDK4/6i treatment and switching, n (%^a^)** | | | | | |
| **1L CDK4/6i + AI** | **Any subsequent treatment**  **n (%)** | PAL-containing | PAL + AI^b^ | RIB-containing | RIB + AI^b^ | ABE-containing | ABE + AI^b^ |
| **1L PAL + AI**  **(n = 6800)** | 3392 (49.9) | 876 (25.8) | NA | 175 (5.2) | 67 (2.0) | 342 (10.1) | 140 (4.1) |
| **1L RIB + AI**  **(n = 2003)** | 748 (37.3) | 152 (20.3) | 115 (15.4) | 142 (18.9) | NA | 105 (14.1) | 73 (9.7) |
| **1L ABE + AI**  **(n = 1441)** | 567 (39.4) | 131 (23.1) | 106 (18.7) | 44 (7.8) | 36 (6.4) | 129 (22.8) | NA |

^a^ Proportion of patients who received any subsequent treatment (defined as a change in systemic therapy triggering a line advancement with the exception of changes from one AI to another).

^b^ CDK4/6i was switched but AI did not change.

Abbreviations: 1L, first-line; ABE, abemaciclib; AI, aromatase inhibitor; CDK4/6i, cyclin-dependent kinase 4/6 inhibitor; NA, not applicable; PAL, palbociclib; RIB, ribociclib; sIPTW, stabilized inverse probability of treatment weighting.

**Supplementary Figure S1. Flowchart of patient selection**


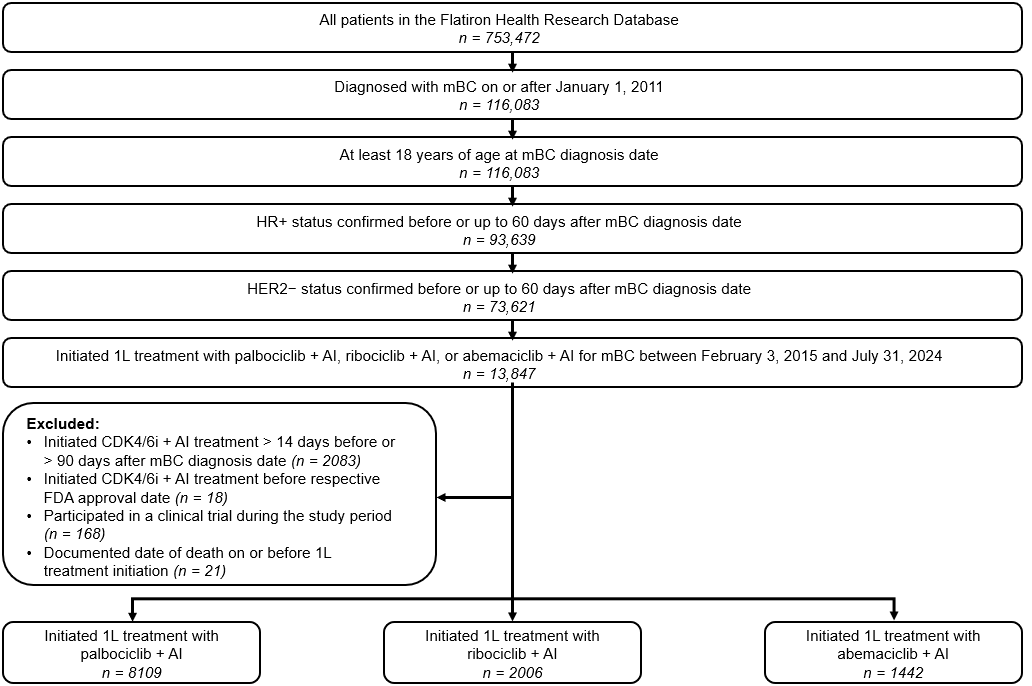


Abbreviations: 1L, first-line; AI, aromatase inhibitor; CDK4/6i, cyclin-dependent kinase 4/6 inhibitor; FDA, US Food and Drug Administration; HER2−, human epidermal growth factor receptor 2-negative; HR+, hormone receptor-positive; mBC, metastatic breast cancer.
